# A preliminary comparison of bellringer performance across three visual modalities for the assessment of anatomy knowledge

**DOI:** 10.1002/ase.70145

**Published:** 2025-10-26

**Authors:** Yiming Zhang, Jeffrey Sun, Kaitlin Marshall, Josh P. Mitchell, Judi Laprade, Joshua P. Nederveen, Peter B. Helli, Irena A. Rebalka

**Affiliations:** ^1^ Education Program in Anatomy McMaster University Hamilton Ontario Canada; ^2^ Division of Anatomy University of Toronto Toronto Ontario Canada; ^3^ Department of Kinesiology McMaster University Hamilton Ontario Canada; ^4^ Department of Nursing McMaster University Hamilton Ontario Canada; ^5^ Department of Pathology and Molecular Medicine McMaster University Hamilton Ontario Canada

**Keywords:** anatomy, assessment validity, bellringer, cybersickness, education, examination, virtual reality

## Abstract

Anatomy education is an inherently visual field, particularly in bellringer (BR) testing, which requires learners to identify anatomical structures on human‐donated specimens. While the traditional use of these physical specimens in BR testing has long remained the standard, three‐dimensional (3D) viewing in virtual reality platforms and two‐dimensional (2D) images of specimens on paper have become common alternatives due to the ease and feasibility of use. Despite widespread use, there is a paucity of literature comparing the assessment validity of these modalities to physical specimens as the historic standard. Thus, this study sought to assess BR testing performance and question validity (using point biserial evaluation) across all three modalities. In total, 140 undergraduate students, enrolled in an Introductory Anatomy and Physiology course at the time of testing, participated in a BR examination with specimens presented in three visual formats: physical specimens, printed 2D images, and 3D reconstructions in virtual reality. When comparing all three modalities, no notable differences were found between question difficulty, point biserial values, presentation of cybersickness, visuospatial ability, or modality preference. Additionally, modality preference and student opinion did not significantly affect test scores, suggesting that these student attributes were unrelated to BR performance. The examinations had high reliability as measured by KR‐20 values, supporting the applicability of our results to undergraduate anatomy BR testing. This study provides preliminary evidence supporting the utility and validity of both 2D images and 3D virtual reality as alternative modalities for BR testing within the undergraduate anatomy education setting.

## INTRODUCTION

Dissection and teaching with human donors are foundational components of anatomy education and have long been the cornerstone of traditional gross anatomical training for learners at all levels, from undergraduates to surgical fellows.^1^, ^2^, ^3^, ^4^ Anatomical donations have also been a staple in all forms of assessments, including bellringer (BR) exams, which have historically provided objective assessments of student knowledge and comprehension of key anatomical concepts.^3^, ^5^, ^6^, ^7^ These assessments have proven challenging in recent years, with increasing critical resource demands for proctors, wet lab space, and large volumes of donated anatomical material, stemming from undergraduate and postgraduate programs experiencing increasing enrollment.^8^, ^9^, ^10^, ^11^ Consequently, there has been considerable growth in the use of alternative testing modalities for BR examinations, including the use of two‐dimensional (2D) photos and three‐dimensional (3D) reconstructions of specimens via virtual reality (VR) platforms.^12^, ^13^, ^14^, ^15^, ^16^ Both alternative modalities have garnered considerable interest in the scientific literature for their use in teaching and learning anatomy; however, investigations of their validity for use within assessments are still warranted.

Although 2D modalities such as computer‐based assessments or printed images are an inexpensive alternative when physical specimens are inaccessible, they result in a loss of visuospatial information concerning the 3D context of anatomy. These factors have been previously shown to impede student response accuracy and disrupt learning outcomes.^17^, ^18^ While monocular cues for perceiving relative size and distance are conserved, a lack of motion parallax and binocular disparity limits an individual's ability to quickly and accurately perceive the shape of a 3D object.^17^, ^18^ Previous studies have also found a higher cognitive effort required to identify structures in 2D compared to physical specimens or 3D reconstructions in VR.^19^, ^20^ Thus, despite the growing popularity of 2D modalities in BR exams, their use must be critically evaluated to ensure that these shortcomings do not interfere with anatomy assessment outcomes.

It is widely understood that the use of anatomical donations in teaching and assessments helps to deepen learners' conceptual understanding and provide a 3D perspective of anatomical structures, which is essential for spatial comprehension of the human body.^21^, ^22^, ^23^, ^24^ With recent technological developments, 3D computer representations of human anatomy have also entered the educational landscape. 3D VR technology has shown success in visualizing educational content in both professional procedural training and contextual learning settings, resulting in improved factual and spatial knowledge acquisition by learners.^25^ Furthermore, VR has become appealing in anatomy teaching and assessment because its setup and maintenance are less resource‐ and cost‐intensive than physical human specimens. Recent studies have shown that learning anatomy within an immersive VR environment improves student engagement, positively influences students' ability to learn and explore anatomy, and enhances classroom learning outcomes compared to alternative teaching methods.^1^, ^5^, ^13^, ^14^, ^22^, ^26^ VR has also received considerable scrutiny regarding its limitations, including user adaptation challenges and cybersickness, a motion sickness‐like phenomenon that occurs while using virtual technologies.^27^, ^28^, ^29^ For VR use in anatomy, the most predominant student‐reported symptoms are eye strain, discomfort, and difficulty concentrating.^5^, ^13^ Previous studies have denied a relationship between cybersickness and examination performance; however, there is uncertainty surrounding its overall impact on the student experience in relation to learning and assessment.^30^ As such, cybersickness remains a key variable requiring further investigation for anatomy educators to better understand the limitations of VR in assessment settings.

Regardless of the testing modality employed, the impact on student experience must be considered when delivering student assessments. Generally, the perceived novelty of a testing modality does not influence test performance.^31^, ^32^ However, studies exploring the use of traditional 2D testing modalities within and beyond anatomy describe a positive relationship between perceived ease of use and test performance.^19^, ^33^ Additional literature also suggests that the effect of preference may be significant in assessments that rely on sensory capabilities, such as within anatomy BR testing.^34^ Therefore, the link between student preference and performance is essential to consider. Though current research indicates that both preference and performance favor the use of physical specimens over virtual 3D models, most students remain open to adopting novel virtual tools.^26^, ^35^ With potential variations in student attitudes towards each modality, understanding the effects of student experiences on performance is critical when designing anatomy examinations.

While the literature highlights the benefits of physical anatomical specimens, 2D representations, and VR in the context of feasibility, educational effectiveness, and learning, there is a paucity of data regarding the use of different modalities for assessing anatomical knowledge. This is especially true for comprehensive and simultaneous comparisons of physical, 2D, and 3D VR specimens in testing anatomical concepts. Researchers and educators alike must better understand the drivers of unfavorable outcomes in examination settings, such as negative student responses and reduced performance when employing different testing modalities, to ensure testing validity.^24^, ^36^ The objective of this preliminary study was to conduct a comprehensive comparison of testing performance and preference between BR examination questions using three different visual modalities: physical specimens, 2D high‐resolution printed photographs of the same physical specimens, and 3D reconstructions of the physical specimens presented in VR. Our primary outcome was question/item difficulty (i.e., the average rate of correct BR responses per modality). Secondary outcomes included point biserial correlations and KR‐20, which measure correlations between individual questions and overall test scores, as well as test reliability, respectively.^37^, ^38^, ^39^ Tertiary outcomes included the evaluation of cybersickness, student experience, and visuospatial ability. Overall, the results of this study will enable educators in the field of anatomy education to make evidence‐based decisions about incorporating appropriate testing strategies into their anatomy curricula.

## MATERIALS AND METHODS

Ethics approval was obtained from the Hamilton Integrated Research Ethics Board (#16591), and all participants provided written informed consent prior to study participation.

### Study participants

In this single‐center study, all undergraduate students registered in a formal first‐ or second‐year Introductory Anatomy and Physiology course at McMaster University (Hamilton, Ontario, Canada) during the Fall 2023 and Winter 2024 semesters were eligible to participate. All anatomy courses covered similar topics and subject matter over the year, using a combination of lectures, labs, tutorials, and BR or alternative assessments. Physical specimens were viewed by these students within biweekly labs, and 2D images were viewed within lectures, labs, and tutorials. While students had the opportunity to engage with VR during tutorials, study participants were not required to possess any previous VR exposure or experience. Although no students had performed a full BR examination prior to this study, students received exposure to BR‐formatted questions during labs and on previous tests. Undergraduate participants were enrolled in the following academic programs: Faculty of Health Sciences (Bachelor of Health Sciences and Integrated Biomedical Engineering) (55% of study participants, 77/140), Bachelor of Science in Nursing (14%, 20/140), Midwifery (3%, 4/140), Faculty of Science (Kinesiology; 27%, 38/140), and Faculty of Engineering (Chemical and Electrical Engineering; 1%, 1/140). All 140 participants completed each study component in its entirety, and no participants or participant data were excluded from any analysis due to incomplete datasets.

### Study design

**FIGURE 1:**
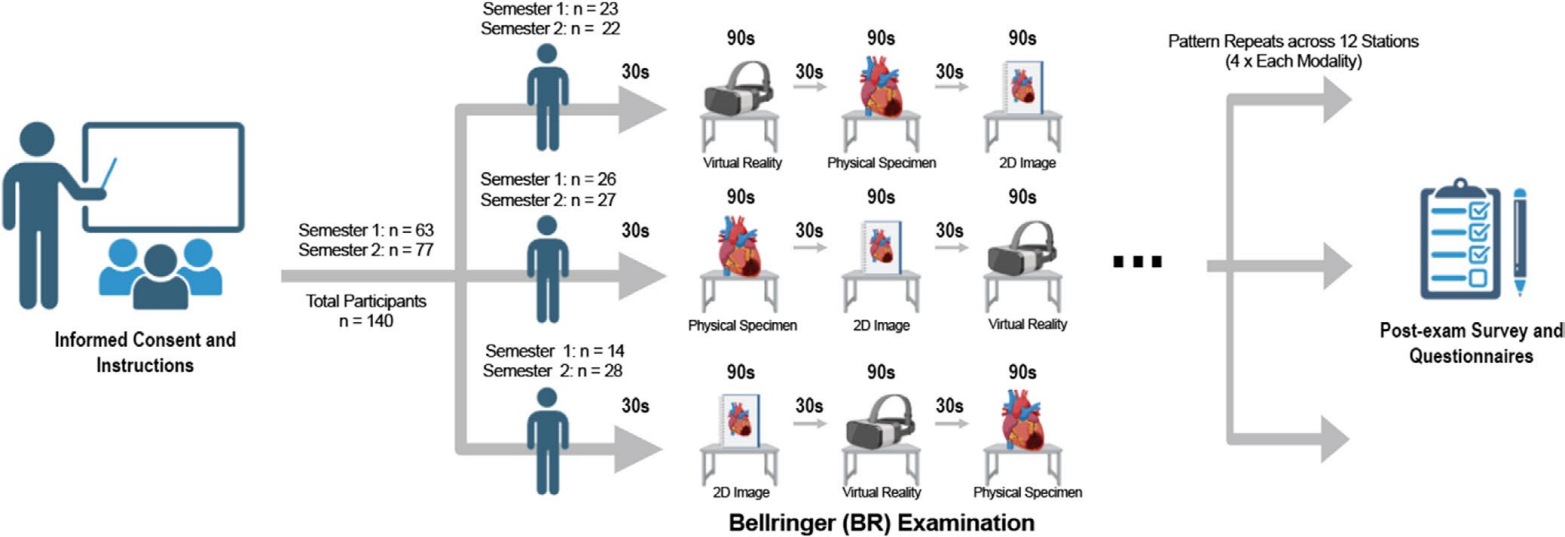
Summary of study protocol, including bellringer exam breakdown. Each semester participated in a unique 12‐question mock bellringer examination comprised of the three rotations specified. Image created using Biorender.com.

A mock BR examination required participants to identify 12 pinned anatomical structures across 12 stations, with one unique specimen per station (Figure 1). Physical specimens were presented on specimen trays. In the 2D representation, a high‐resolution printed photograph of the same specimen was presented on paper. Each photograph provided a full specimen view to ensure that context was maintained. These 2D images were photographed using a high‐resolution camera (Sony Alpha a6300; Sony; Tokyo, Japan) and color‐printed on 8.5″ × 11″ cardstock. In VR, a 3D reconstruction of the same specimen was presented. A high‐definition 3D light scanner (Space Spider; Artec 3D; Senningerberg, Luxembourg) and its complementary software (Artec Studio 17; Artec 3D) were used to create 3D file images for post‐processing (via Blender; Blender Institute; Amsterdam, Netherlands). To mimic a true BR test in VR, 3D images were viewed by participants in the Meta Quest 2 (Meta; Cambridge, MA, USA) VR headset device in a program specially created for the study in the Godot game engine (version 3.4; Godot) at a native resolution of 1832 × 1920 pixels per eye. Specimens were presented at a scaled size, and students were given only the freedom of motion of their headset, with no interactive capabilities, to preserve an authentic examination experience. Study participants were not allowed to use handheld controllers during the trial. To ensure that each question was consistently represented across all three visual modalities, analogous stations across three distinct rotations presented the same 12 BR questions in all three visual modalities based on the rotation circuit. With a total of 12 questions per rotation, each of the three visual modalities appeared four times per rotation to each student.

Participants were distributed based on logistical availability. Reflecting undergraduate and postgraduate BR testing at McMaster University, participants were given 90 s to observe each specimen and provide a response to the associated question prompt. Additionally, 30 s of transition time allowed movement to the next station within the rotation. Participants self‐selected their starting station and completed one rotation each. Students were instructed to treat their participation as a legitimate examination and were proctored by investigators for the duration of the examination. The total duration of the BR trial was 25 min (112 stations, with 90‐s question time and 30‐s transit time each). Upon completing the trial, students were given a series of questionnaires to complete. Independent versions of the BR trial, containing 12 different specimens and questions each, were offered in December 2023 and April 2024, totaling 24 questions. Questions represented content learned over the Fall (December 2023) or Winter (April 2024) semester to mimic a true test‐taking scenario.

### Data collection

#### Bellringer responses

For each question, students were required to input an anatomical structure as a short, free‐recall response. Within the BR exam, one point was awarded for each correct response (i.e., a correct identification of the labeled specimen), and zero points were awarded for any incorrect or partially correct attempt. No word bank or suggestions were provided. The maximum score achievable was 12 points.

#### Study‐specific questionnaire, simulation sickness questionnaire, and mental rotations test

Immediately following the BR, participants completed three single‐measure questionnaires on paper: a general, study‐specific questionnaire, the Simulation Sickness Questionnaire (SSQ), and the Mental Rotations Test (MRT). All three questionnaires can be found appended to this manuscript within the supporting information attachment (Figures S3 and S4).

The study‐specific questionnaire contained categorical scales concerning personal preferences and experiences with each of the three modalities. This questionnaire also explored previous VR experiences and the use of glasses or contact lenses.

The SSQ, a 16‐item scalar qualitative scale to rate cybersickness symptoms, was quantitatively summed and assessed on a scale of 0–3, with “none” representing zero and “severe” representing the maximum of 3. The maximum possible score for the SSQ was 48, or a selection of “severe” for all 16 items on the scale.^40^ The SSQ, originally applied in the context of U.S. Navy flight simulators, is widely used to evaluate cybersickness due to its comprehensive symptom inclusion and validation for VR simulation. Given the limited motion of the VR BR testing environment, the SSQ was deemed an appropriate evaluation for the current VR trial.^41^ Cronbach's alpha for this SSQ was previously reported as adequate (>0.80) among post‐secondary students and the general adult population.^42^, ^43^ The original SSQ, alongside an updated method of analysis, was used herein.^44^

The MRT asked participants to match rotated versions of a unique shape to a template shape. There were 20 questions in total. Following the original MRT instructions piloted across all levels of education, a 10‐min time limit was allotted for this test.^45^ The maximum possible score for the MRT was 20, or one point per correctly answered question. The MRT has been demonstrated to possess strong internal validity, with a Cronbach's alpha of 0.91 for undergraduate students and high test–retest reliability.^45^, ^46^, ^47^

Completion of all surveys was quasi‐voluntary, whereby participants may have felt obligated to complete the surveys due to known ties to the study's lead investigator (IAR), a faculty member within the McMaster University Education Program in Anatomy. Notably, she was not present for the administration of the BR exam or completion of the surveys. No grade‐based incentives were provided, and no instructors were made aware of student participation in this study. Participants received monetary compensation for their participation in the trial. No survey was sponsored by external bodies. Survey completion was supervised and tied to unique participant identification numbers to minimize response bias.

### Data analysis

Only participants who completed both the BR examination and the associated post‐examination questionnaires were included in the dataset for analysis. Since the results from the two experimental trials did not differ significantly, all data from December 2023 and April 2024 were combined for reporting purposes. Error was reported as the standard error of the mean (SEM) or in the form of a 95% confidence interval (CI), as denoted within each figure caption. Given that the student performance data passed Levene's test of equal variance and normal Q‐Q plotting, one‐ and two‐way analysis of variance (ANOVA) tests were used to detect group differences. The alpha level was set at 0.05. When ANOVA tests reported significant results (*p* < 0.05), Tukey's multiple comparison test was employed to detect individual group differences. Statistical tests were performed using GraphPad (version 10.2.3; GraphPad Software Inc.; San Diego, California, USA) and R Studio (version 4.3.2; R Core Team; Vienna, Austria).

#### Item difficulty

Item difficulty gauges the average score of participants within a particular question or modality. This was calculated by dividing the sum of correct BR answers by the total number of questions administered.

#### Point biserial correlations

The point biserial correlation is a discrimination index that gauges the ability of questions to discriminate between participants' performance. In item analysis, it is a statistical measure that calculates the relationship between a test taker's score on a single dichotomous item (correct or incorrect answer) and their overall test score, indicating how well that specific item differentiates between high‐ and low‐performing test takers. It is a classical test theory approach for assessing the discriminating power of an individual test question within a larger assessment. A high point biserial indicates that participants who answered a specific BR question correctly also performed well on the test overall. Average point biserial values were calculated for each modality across all 12 items. A threshold of point biserial >0.30 was used to determine a successful question.^38^, ^48^, ^49^

#### Reliability analysis

The Kuder–Richardson Formula 20 (KR‐20) is a correlational measure that reports the consistency of test‐takers' responses to a test with dichotomous outcomes.^50^ A high KR‐20 indicates that the test is a homogeneous pool of items with a common focus, indicating higher reliability. The KR‐20 is influenced by the difficulty, spread, and length of the test, and is suited to situations when questions vary in difficulty.^39^ The strength of KR‐20 was reported based on DeVellis's parameters, wherein: <0.60 was unacceptable, 0.60–0.65 undesirable, 0.65–0.70 minimally acceptable, 0.70–0.80 is respectable, 0.80–0.90 very good, and >0.90 excellent.^39^

#### Convergent validity

Pairwise Pearson's correlations between student sub‐scores on Physical, 2D, and VR bellringer items were calculated to evaluate convergent validity across all three modalities.

#### Study‐specific questionnaire

A one‐way ANOVA was performed on all categorized data, including participant modality preferences related to participant test performance. A two‐way ANOVA was performed with preference and SSQ score ranges as covariates, and they were compared to the VR BR test results.

#### Simulation Sickness Questionnaire and Mental Rotations Tests

The relationship between participant characteristics and their SSQ scores was analyzed by simple linear regression. Pearson's coefficient of determination (*R* ^2^) was also calculated. Linear plots show standard error, while categorical plots show sample mean ± SEM. Participants' success on VR questions and the cumulative sum of their SSQ scores were analyzed by one‐way ANOVA. These analyses were repeated using MRT scores, with one‐way ANOVA analyses comparing cumulative MRT scores with VR BR scores, total BR scores, and SSQ scores.

## RESULTS

### Question difficulty was unaffected by visual modality, while discriminatory capacity differed between VR and physical specimens

**FIGURE 2:**
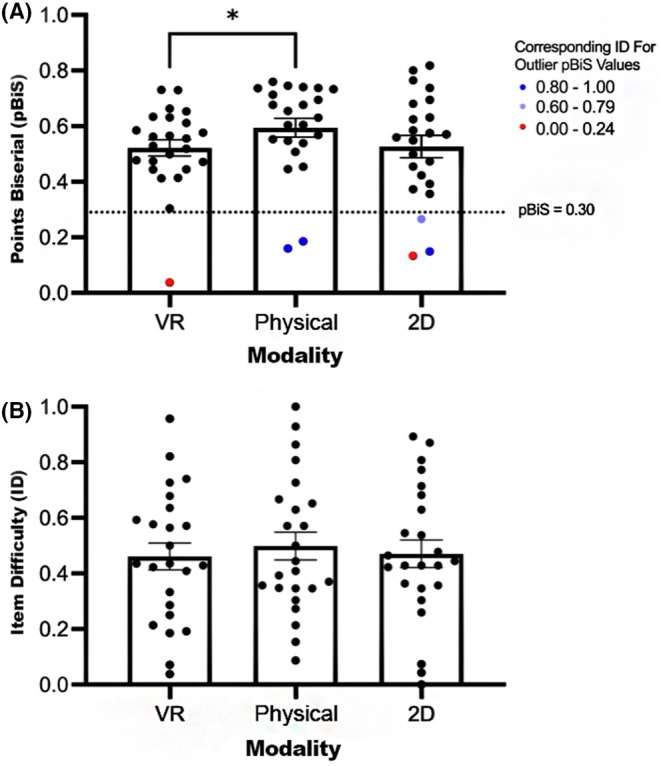
Question difficulty was unaffected by visual modality, but discriminatory capacity (point biserial) differed between presentation in VR versus physical specimens. (A) Overall comparison of point biserial distributions between modalities. Legend represents corresponding item difficulty for each question with a point biserial below 0.30, indicating poor question discriminative quality. (B) Overall comparison of item difficulty distribution between modalities, including invalid question data. Mean and individual data points are presented in each panel. Error bars represent SEM. Asterisk (*) represents statistically significant differences (*p* < 0.05) detected by one‐way ANOVA analysis following the removal of questions with a point biserial value <0.30.

No statistically significant differences were observed for point biserial values between the three modalities. However, after removing poorly performing questions with point biserials <0.30 (Figure 2A), a significantly higher (*p*‐value = 0.0053) point biserial value for physical questions (point biserial = 0.6342 ± 0.0217) was detected compared to VR (point biserial = 0.5427 **±** 0.0222). No statistically significant differences were present between modalities with respect to item difficulty after excluding poorly performing questions. Therefore, all questions from all modalities were included in subsequent analyses (Figure 2B). Item difficulty and point biserial means are reported in Table S1, both including and excluding the six questions (VR *n* = 1; physical *n* = 2; 2D *n* = 3) considered invalid with point biserial values <0.30.

### Additional testing parameters revealed reliability and convergent validity across modalities

KR‐20 for BR exams held in December 2023 and April 2024 were 0.736 and 0.830, respectively, with an average of 0.785 across both BR exams. These KR‐20 values were above the threshold of acceptable reliability.^39^

Pearson's correlation coefficient (*r*) revealed moderate significant relationships for all pairwise performance comparisons (VR to 2D *r* = 0.487, *R* ^2^ = 0.237, *p* < 0.001; VR to physical *r* = 0.522, *R* ^2^ = 0.273, *p* < 0.001; physical to 2D *r* = 0.5219, *R* ^2^ = 0.272, *p* < 0.001), providing evidence of convergent validity among the tested modalities. Low *R* ^2^ values imply that additional modality‐specific factors are influencing test results.

### Reports of cybersickness were minimal and did not affect VR or total BR test scores

The maximum possible score for the SSQ was 48, representing the maximum degree of cybersickness symptoms. SSQ scores were stratified into four discrete categories: negligible (SSQ score under 4), minimal,^5^, ^6^, ^7^, ^8^, ^9^ significant,^10^, ^11^, ^12^, ^13^, ^14^ and concerning (≥15).^51^ SSQ scores were predominantly negligible and minimal, with 89% (124/140) of participants possessing an SSQ score < 10. Only 2% (3/140) of participants had a “concerning” SSQ score, with the highest reported score being 23. The most commonly reported symptoms were difficulty focusing (67%, 95/140), blurred vision (62%, 87/140), and eyestrain (61%, 86/140). No statistically significant differences in SSQ scores were observed despite stratifying between student attitudes toward VR (Figure S1A), student use of glasses or contacts (Figure S1B), previous VR experience (Figure S1C), or perceived comfort with VR (Figure S1D).

No significant relationships were observed between total SSQ scores and overall BR success or VR question success (Figure S2A,B). To ensure diligent evaluation, the 16 items of the SSQ questionnaire were divided into two categories, namely nausea and oculomotor symptoms.^44^ Once again, no correlations were found between total SSQ scores and student VR BR success (Figure S2C,D).

### Visuospatial ability was weakly associated with overall BR and VR question success, while possessing NO relationship with reported cybersickness

**FIGURE 3:**
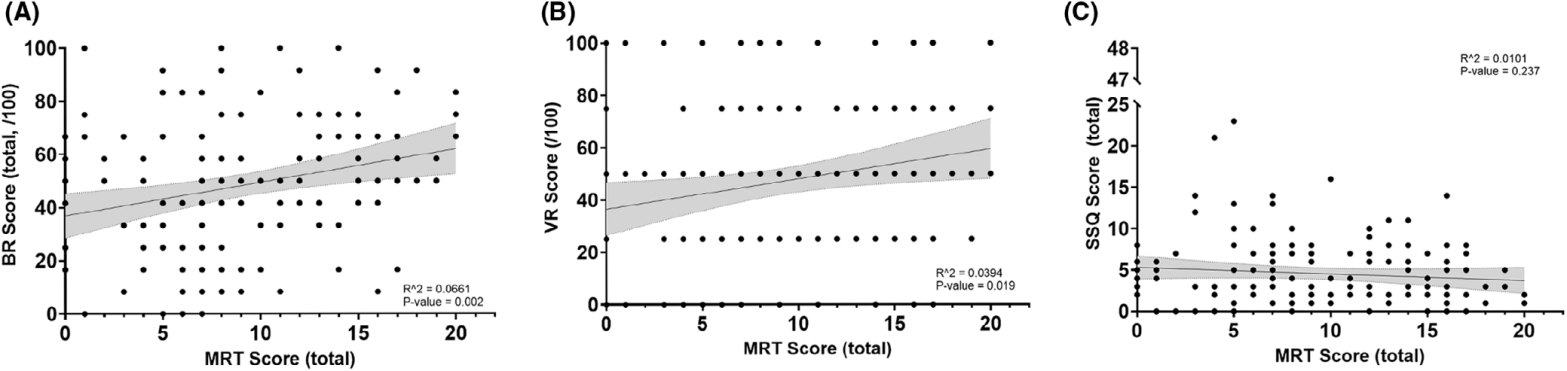
Mental rotation test (MRT) scores were not correlated with bellringer performance or cybersickness severity. (A) MRT score compared to total BR success and (B) MRT score compared to VR question success showed significant but negligible correlations. (C) There was no correlation between MRT score and cybersickness score. Shaded area represents the 95% CI. Significance was set at *p* < 0.05.

Significant, though negligible (*p* < 0.05, *r* ^2^ < 0.1), positive linear relationships were observed between MRT and BR scores (Figure 3A; *R* ^2^ = 0.0661, *t* = 3.03, *p* = 0.002) as well as between MRT and VR BR scores (Figure 3B; *R* ^2^ = 0.0394, *t* = 2.38, *p* = 0.019). There was no relationship between MRT and SSQ scores (Figure 3C; *R* ^2^ = 0.0101, *t* = 2.38, *p* = 0.237).

### Participants strongly preferred the use of physical specimens; however, preference did not affect exam performance

As outlined in Table 1, 62% (87/140) of students preferred answering questions using physical specimens, and 50% (70/140) had the most confidence answering questions using physical specimens. By comparison, 54% (76/140) of students reported that VR was their least preferred modality, and 50% (70/140) of students had the least confidence answering VR questions. Physical specimens were noted as the easiest format to use (50%, 70/140), while VR was the most difficult (74%, 104/140). When asked to categorize modalities by the following descriptor, “It was clearest to understand what I was looking at in this format,” 64% (90/140) of participants chose physical specimens.

**TABLE 1:** Bellringer modality preference of study participants.

Rating statement	Modality			
	VR	Physical	2D	Other/NA
I *most* preferred answering questions in this format	21	87^a^	29	3
I *least* preferred answering questions in this format	75^a^	13	50	2
I was *most* confident answering questions in this format	16	70^a^	53	1
I was *least* confident answering questions in this format	70^a^	30	48	2
I found this format *easiest* to use	8	70^a^	61	1
I found this format *most difficult* to use	103^a^	9	27	1
It was *easiest* to recall information I know in this format	20	68^a^	50	2
It was *hardest* to recall information I know in this format	77^a^	23	37	3
It was *clearest* to understand what I was looking at in this format	18	90^a^	21	1

^a^ Denotes the most popular response for each question. One hundred forty students answered each question.

Despite an overarching preference for physical specimens over both VR and 2D and disinclination for VR, when asked, “How interested are you in seeing VR used in anatomy examinations within your courses at McMaster University?” 71% (99/140) of participants were in support or neutral, whereas only 29% (41/140) were against its use.

**FIGURE 4:**
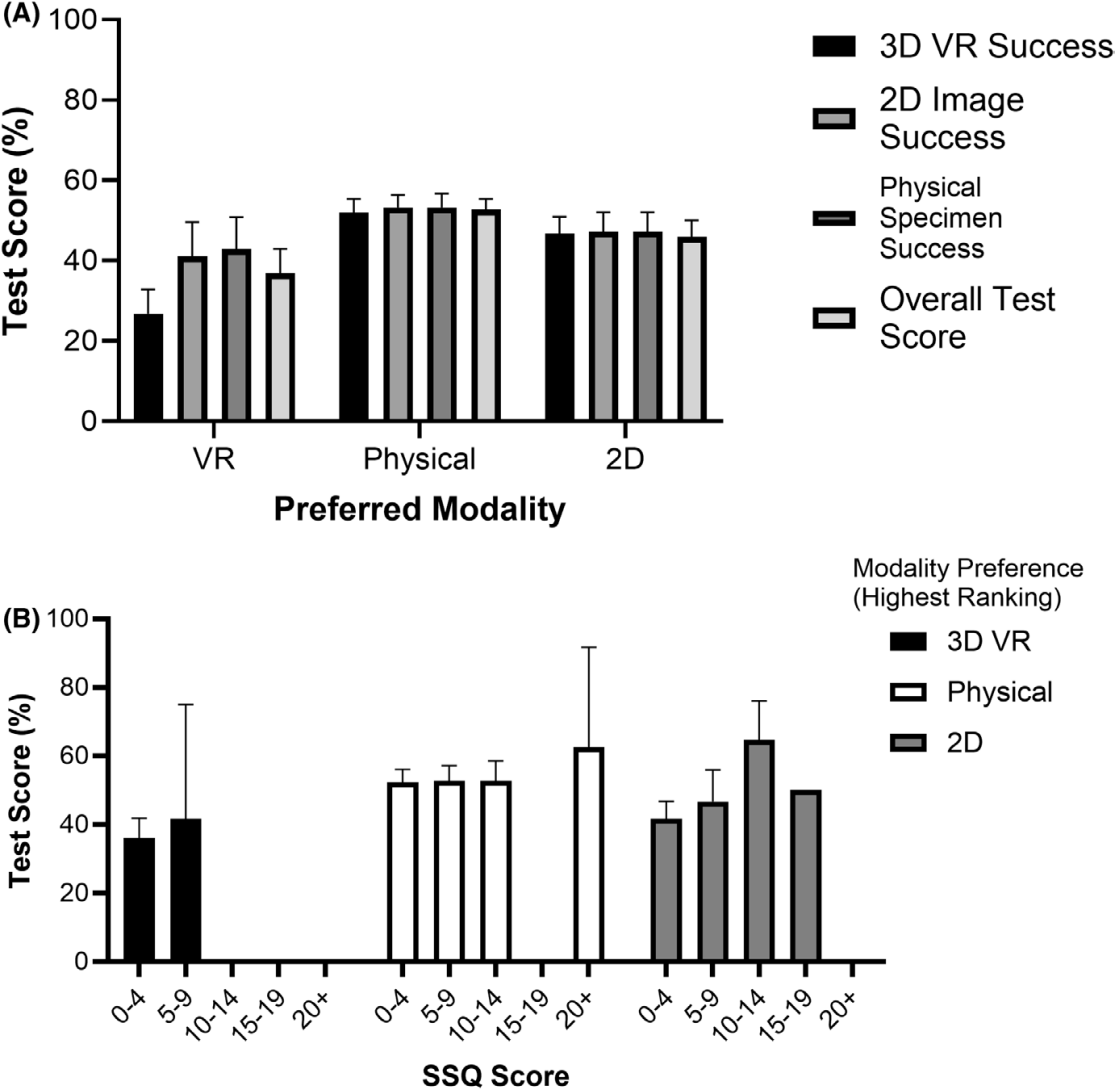
Bellringer exam score was not affected by modality preference or cybersickness severity. (A) Average test performance stratified by preferred testing modality. (B) Average test performance stratified by preferred testing modality as well as cybersickness score range. Bars represent means, while error bars represent SEM. No significant differences were found via one‐ and two‐way ANOVA.

One‐way ANOVA was conducted to explore the relationship between test success and modality preference. Despite a significant *p*‐value (*p* = 0.045), the effect size was weak (*R* ^2^ = 0.0449). To further explore this phenomenon, no significant differences were observed in Tukey's post‐hoc analysis (Figure 4A). Further stratifying data by SSQ score yielded no relationship between preference and performance via two‐way ANOVA (Figure 4B; *p* = 0.682).

## DISCUSSION

Creating and validating examinations that accurately assess student knowledge provides instructors with comprehensive feedback for tailoring future curricular components and fostering student success.^52^, ^53^ This study is among the first to compare physical specimens, 2D, and 3D VR modalities in an undergraduate anatomy BR setting. Overall comparisons between item difficulty and point biserial values found no statistically significant differences between the three visual modalities. As illustrated in Figure 2, the removal of poorly performing question items (with point biserial values <0.30) revealed some discrepancy between the average discriminatory power of VR (point biserial = 0.543) and physical specimens (point biserial = 0.634). However, both values were above the accepted threshold for item quality, and thus, we cannot claim superiority of one modality over another. Additionally, point biserial averages yielded no statistically significant differences when poorly performing questions were included. Similar results were observed with item difficulty comparisons when invalid questions were included. Despite containing a few ‘unsatisfactory’ questions, the examinations for all three modalities had strong item‐level psychometric properties and respectable reliability values. Of note, the consistent moderate to weak correlations observed between sub‐scale performance provide evidence of convergent validity across modalities and suggest that all three modalities assess anatomical knowledge to a similar extent. However, there is a possibility that the small sample size (four questions per visual modality per test) may underestimate true convergent validity, and modality‐specific factors may still influence performance. An extension of this work, which includes more questions per visual modality, will help refine these estimates and further clarify construct overlap.

It was hypothesized, based on previous literature, that 2D testing would lead to inferior student performance due to the removal of 3D visual cues and stereopsis. However, testing performance did not differ across the three testing modalities in terms of item difficulty or point biserial. It is possible that the high‐quality 2D images of specimens used in this trial provided more visual information, resulting in test performance scores that were no different from those of the other two testing modalities. While appropriate for BR testing purposes, further connections between the limitations of visual clarity and test performance should be resolved to more definitively explore the limitations of 2D image‐based anatomy assessments.

Exploring the implications of this study for the use of VR in anatomy settings is of critical importance. Regarding the potential influence of cybersickness on academic performance, these trial results align with those previously reported by Brewer‐Deluce et al. and Saraco et al., whose participants also reported a high incidence but low severity of cybersickness using VR.^5^, ^13^ While MRT scores showed a statistically significant positive correlation with both VR and overall BR test success, the low *R* ^2^ values (VR *R* ^2^ = 0.0394; BR *R* ^2^ = 0.0661) indicated that the correlation was negligible. Given the absence of a relationship between SSQ or MRT scores with VR question performance, neither cybersickness nor visuospatial ability posed a significant detriment to student success during this trial.

Cybersickness has previously been shown to negatively affect visuospatial working memory and psychomotor skills, which can negatively impact student cognition and recall in examination settings, resulting in poor test performance.^54^, ^55^ In the current study, participants reported low SSQ scores, with 89% (124/140) of students experiencing minimal or negligible cybersickness symptoms (SSQ < 10), suggesting that VR can be employed in short‐term settings, such as a BR exam, without negative consequences. Cybersickness is thought to be correlated with increased VR usage duration, with reports of nausea and disorientation lower for exposure times of <10 min versus those >10 min.^56^, ^57^, ^58^, ^59^, ^60^ Paradoxically, individuals who use VR for more than 20 min experience fewer symptoms than those who use it for 10–20 min.^60^ As mentioned previously, each student was provided 90 s per station, in accordance with historical testing protocols at McMaster University for BR exams, for a theoretical maximum of 6 min of VR exposure over the course of the trial. This was done to provide an institutionally authentic BR testing experience. While our current data contradict several studies that describe negative consequences of cybersickness on testing performance, these consequences may still exist and may only be apparent with longer VR exposure timeframes.^12^, ^55^, ^61^ Similarly, although not correlated with overall student performance, visuospatial ability was lower than expected based on previous reports.^62^, ^63^ The significant yet weak interaction between visuospatial ability and student VR BR performance, however, contradicts recent literature that found a positive relationship between MRT score and VR anatomy examination performance.^12^, ^55^, ^61^ Although speculative due to limitations in our current methodology, including the sparse number of questions to which students were exposed, the brevity of the VR experience in this investigation may have led to lower VR contact times and application of visuospatial abilities.

Several previous studies noted significantly higher examination scores among anatomy students who used VR to study compared to those who used non‐VR modalities, with a focus on the study process over assessment delivery.^13^, ^23^, ^36^, ^55^ The current trial found that VR use may not significantly improve educational outcomes in all contexts, despite a lack of contraindication from cybersickness and differences in student performance related to MRT scores. Methodological differences may be responsible for these variations, although the average KR‐20 value across the exams supports the overall reliability of the exam. Current data indicate that VR may not be superior for all testing purposes and, as such, the use of physical specimens or 2D images for testing anatomy knowledge will likely continue to be the standard at many institutions. These findings lessen the immediate fiscal pressure on educators and their respective institutions to invest in VR over standard images.

We observed no correlation between modality preference and exam performance. Furthermore, even though a majority of participants selected VR as their least preferred testing modality, there was equal selection for supporting (*n* = 22) or being against (*n* = 23) VR use for BR exams, with a slightly greater number of students (*n* = 30) being ‘neutral’ regarding VR's future application in anatomy testing. This preference aligns with earlier studies, yet it simultaneously contradicts a common claim in prior research that VR preference is correlated with improved VR performance.^31^, ^64^, ^65^ Our results suggest that preference was influenced by external factors unrelated to a student's ability to excel at any given test modality. Previous work aligns with this notion, wherein student preference for an examination delivery method reflects perceived difficulty or familiarity, irrespective of true performance capability.^66^, ^67^, ^68^

## LIMITATIONS

A fundamental consideration in experimental designs is the need for sufficient statistical power to support meaningful interpretation of outcomes. This requires not only an adequate participant sample size but also a robust number of test items within each modality under investigation to detect significant effects or to yield generalizable conclusions. While our overall sample size was sufficient (*n* = 140), per modality reliability estimates were based on a limited sample (four questions per modality per test), which inflates sampling error and reduces the stability of KR‐20. Therefore, these values should be taken as exploratory pilot metrics. Future studies employing larger item pools per modality will be essential to confirm these preliminary findings and strengthen generalizability.

Additionally, demographic data, including sex and age, were not collected from study participants. As such, potential demographic variations in modality success were not explored. Moreover, while students in each semester received the same test questions during this investigation, the BR exam content between December 2023 and April 2024 differed since different body systems were explored in lecture as the terms progressed; for example, neuroanatomy and the musculoskeletal system were covered in the fall term, and cardiovascular anatomy was covered in the winter term. Therefore, each BR mock exam reflected those topics, respectively. This was done to match the content of the BR evaluation with the content being taught during the semester in which students participated in this trial. When exclusively analyzing items with point biserial values <0.30, it was found that questions with lower item difficulty (i.e., harder items) exclusively tested central nervous system structures. In contrast, those with higher item difficulty (i.e., easier items) tested major organs and muscles. Additionally, lower item difficulty (i.e., harder) questions were predominantly tested in the VR modality, whereas easier questions were mainly evaluated using physical specimens. Item difficulty was consistent across each session and modality. However, this suggests the possibility that certain anatomical structures may be better represented by specific modalities and that item content may influence test outcomes. Given that the current study did not examine modality performance within specific anatomical themes or organ systems, the answer to this question remains unclear.

Test timing may also be noteworthy, given that the 90 s students were given to answer BRs may have been too short for adequate cybersickness to arise.^69^, ^70^ While a study by Woo et al. demonstrated that, upon symptom onset, an average recovery time from VR sickness was roughly 12 min, previous studies have argued that cybersickness onset and recovery vary considerably with the length and style of the VR experience.^29^, ^71^, ^72^, ^73^, ^74^ With the current trial mirroring a test‐taking setting, uneven or rigorous movements were sparse, and with low exposure time, results may theoretically vary in longer test‐taking settings. Furthermore, questions may be raised about whether the emotional state of the test taker could interfere with the onset of cybersickness in a true BR examination setting, as compared to the current study's emulation of one. Therefore, our SSQ data may not fully represent future scenarios where VR is used for entire examinations.

Future investigations that control question content and reflect the realistic application of VR in an examination may provide more data on the benefits of VR and susceptibility to cybersickness in education. A greater understanding of cybersickness, and the subsequent control of its effects, will be essential for developing future trials that explore the use of VR modalities in testing and education.

## FUTURE DIRECTIONS

It is essential to acknowledge that neither the testing modality nor student preference affected test performance. Educators should therefore consider their examination goals when employing novel modes of evaluation. For example, testing using 2D images may help reduce expenses compared to VR and physical modalities, while having no adverse impact on testing performance. VR may also be a viable alternative testing option for remote education, with negligible differences between it and other common modalities. The lack of significance between modality preference and testing performance suggests that student performance will remain consistent regardless of shifting attitudes toward incoming novel modalities. We also introduce the prospect that specific modalities may be more suitable in certain scenarios. If trends seen among poorly performing questions hold true, physical specimens may be more suitable for representing fine structures whose details may be diminished by the VR rendering process or 2D imaging, which strips much of the 3D context. Finally, though not explicitly investigated in the current study, the modality type and duration of use by students while in class or studying may also impact modality performance during testing. Transfer‐appropriate processing (TAP) is the notion that students score higher on examinations if the environments or modalities used across testing and studying align.^9^, ^75^ While the opportunity to engage, learn, and study with all three visual modalities was available to students, the study methods employed by students were not investigated or monitored within the current study. TAP may help explain discrepancies in testing performance observed between students. Future studies exploring the use of these three modalities for testing purposes must consider how the outcome is influenced by TAP to provide more holistic evaluations.

Beyond advocating for further testing of all three modalities in other anatomical examination contexts, additional study of the VR modality will create more robust data regarding the onset and cumulative effects of cybersickness on the student experience. Further exploring recovery time from cybersickness symptoms is equally imperative to understand its impact on student learning and assessment in all VR environments. To ensure participant characteristics are standardized, researchers may find it valuable to collect data on students' study habits and final examination results to create a baseline performance for each student prior to data analysis. Researchers should be aware that the risk of cybersickness interference may vary depending on the educational context. Future exploration of the diverse applications of the three evaluated visual modalities in examination settings is encouraged.

## CONCLUSION

This preliminary investigation provided insight into the comparability of using physical specimens, VR, and 2D images for anatomical bellringer testing. Results demonstrated no statistically significant differences in average item difficulty or point biserial values between modalities. Student modality preference did not correlate with examination performance. Moreover, examination performance was not correlated with cybersickness or visuospatial ability. Educators should recognize that these results support the application of either 2D imaging or VR modeling as viable alternatives to the standard human‐donated specimens for future bellringer examinations. Nevertheless, further research is needed to investigate the strengths and limitations of alternative testing modalities for accurately assessing diverse anatomical topics.

## AUTHOR CONTRIBUTIONS

**Yiming Zhang:** Writing – original draft; writing – review and editing; investigation; formal analysis; visualization; data curation; methodology; validation. **Jeffrey Sun:** Investigation; formal analysis; writing – review and editing; writing – original draft; visualization; data curation; methodology; validation. **Kaitlin Marshall:** Methodology; writing – review and editing; resources; software; investigation. **Josh P. Mitchell:** Writing – review and editing; software; investigation; methodology; resources; conceptualization. **Judi Laprade:** Writing – review and editing; software; resources; supervision. **Joshua P. Nederveen:** Writing – review and editing; conceptualization. **Peter B. Helli:** Writing – review and editing; conceptualization; methodology. **Irena A. Rebalka:** Supervision; methodology; conceptualization; funding acquisition; validation; investigation; writing – review and editing; data curation; project administration; resources; visualization.

## FUNDING INFORMATION

This study was funded by the Education Services of the Faculty of Health Sciences at McMaster University and the McMaster Education Research Innovation and Theory Education Scholarship Fund.

## CONFLICT OF INTEREST STATEMENT

None to declare.

## ETHICS STATEMENT

This study was approved by the Hamilton Integrated Research Ethics Board (#16591).

## Supporting information


Data S1.
