# Supplementary material for: A preliminary comparison of bellringer performance across three visual modalities for the assessment of anatomy knowledge
Source: Anat Sci Educ. 2025 Oct 26;19(1):72–84. doi: 10.1002/ase.70145 (PMC12748052; doi:10.1002/ase.70145)
Supplement: Supplementary file 1 — Data S1. [file ASE-19-72-s001.docx]

**SUPPLEMENTAL MATERIAL**





**Supplemental Figure 1.** Total cybersickness (SSQ) scores were not affected by (A) attitude towards the virtual reality platform, (B) use of glasses or contacts, (C) previous experience with VR, or (D) user’s personal perceived comfort with virtual reality (VR). Mean ± SEM is reported. No significant differences were revealed via one-way ANOVA.





**Supplemental Figure 2.** Total cybersickness (SSQ) scores were not significantly correlated with (A) overall bellringer (BR) exam performance or (B) virtual reality (VR) question performance during the BR exam. When investigated independently, categorical cybersickness scores for (C) nausea and (D) oculomotor symptoms still showed no significant correlation with VR question performance during the BR exam. Shaded area represents 95% CI.

Thank you for completing the **Comparison of three visual modalities for the assessment of anatomy knowledge (VISAA)** bellringer exam! Please complete this questionnaire in its entirety to complete your participation in the VISAA study.

VISAA STUDY ID: __________

The following set of questions are asking you to compare the three visual modalities that you used today – **in-person** (cadaveric specimens), **2D** (paper images), and **3D** (VR headsets).

|  | | | **In-person** | | | | **2D** | | | **3D** | | |  |
| --- | --- | --- | --- | --- | --- | --- | --- | --- | --- | --- | --- | --- | --- |
| I *most* preferred answering questions in this format: | | | ⃝ | | | | ⃝ | | | ⃝ | | |  |
| I *least* preferred answering questions in this format: | | | ⃝ | | | | ⃝ | | | ⃝ | | |  |
| I was *most* confident answering questions in this format: | | | ⃝ | | | | ⃝ | | | ⃝ | | |  |
| I was *least* confident answering questions in this format: | | | ⃝ | | | | ⃝ | | | ⃝ | | |  |
| I would most prefer taking an entire bellringer exam in this format: | | | ⃝ | | | | ⃝ | | | ⃝ | | |  |
| I found this format *easiest* to use: | | | ⃝ | | | | ⃝ | | | ⃝ | | |  |
| I found this format *most difficult* to use: | | | ⃝ | | | | ⃝ | | | ⃝ | | |  |
| It was *easiest* to recall information I know in this format: | | | ⃝ | | | | ⃝ | | | ⃝ | | |  |
| It was *hardest* to recall information I know in this format: | | | ⃝ | | | | ⃝ | | | ⃝ | | |  |
| It was clearest to understand what I was looking at in this format: | | | ⃝ | | | | ⃝ | | | ⃝ | | |  |
| Do you have any previous experience with VR headsets? | | ⃝ Yes | | ⃝ No | |  | | | | | | | |
| If yes, in what setting? | ⃝ Home / recreation | | | | | | | ⃝ School / education | | | | | |
| How comfortable are you with virtual reality headsets? | | ⃝ 1 (I am not) | | | ⃝ 2 | | | | ⃝ 3 | | | ⃝ 4 (Expert) | |
| Did you wear contacts or glasses during the bellringer exam? | | ⃝ No | | ⃝ Yes - contacts | | | | | | | ⃝ Yes - glasses | | |
| How interested are you in seeing VR used in anatomy examinations within your courses at McMaster University? | | ⃝ Against this | | | | ⃝ Neutral | | | | | ⃝ Support this | | |

Did you experience any of the following symptoms while using the VR headsets? Please check the column that applies to you for each symptom:

|  | **None** | **Slight** | **Moderate** | **Severe** |
| --- | --- | --- | --- | --- |
| General Discomfort | ⃝ | ⃝ | ⃝ | ⃝ |
| Fatigue | ⃝ | ⃝ | ⃝ | ⃝ |
| Headache | ⃝ | ⃝ | ⃝ | ⃝ |
| Eyestrain | ⃝ | ⃝ | ⃝ | ⃝ |
| Difficulty Focusing | ⃝ | ⃝ | ⃝ | ⃝ |
| Increased Salivation | ⃝ | ⃝ | ⃝ | ⃝ |
| Sweating | ⃝ | ⃝ | ⃝ | ⃝ |
| Nausea | ⃝ | ⃝ | ⃝ | ⃝ |
| Difficulty Concentrating | ⃝ | ⃝ | ⃝ | ⃝ |
| Fullness of head | ⃝ | ⃝ | ⃝ | ⃝ |
| Blurred vision | ⃝ | ⃝ | ⃝ | ⃝ |
| Dizziness (eyes open) | ⃝ | ⃝ | ⃝ | ⃝ |
| Dizziness (eyes closed) | ⃝ | ⃝ | ⃝ | ⃝ |
| Vertigo | ⃝ | ⃝ | ⃝ | ⃝ |
| Stomach awareness | ⃝ | ⃝ | ⃝ | ⃝ |
| Burping | ⃝ | ⃝ | ⃝ | ⃝ |

**Supplemental Figure 3.** Co-delivered study-specific questionnaire and cybersickness questionnaire (Simulator Sickness Questionnaire).

**Study ID:** *__________________________*

**M.R.T Test**

This is a test of your ability to look at a drawing of a given object and find the same object within a set of dissimilar objects. The only difference between the original object and the chosen object will be that they are presented at different angles. An illustration of this principle is given below, where the same single object is given in five different positions. Look at each of them to satisfy yourself that they are only presented at different angles from one another.


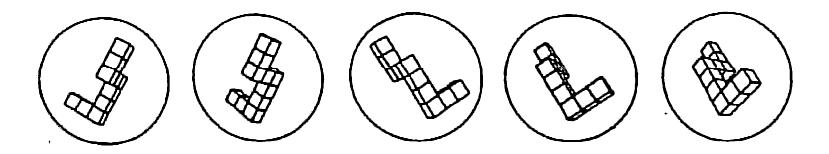


Below are two drawings of new objects. They cannot be made to match the above five drawings. Please note that you may NOT turn over the objects. Satisfy yourself that they are different from the above.


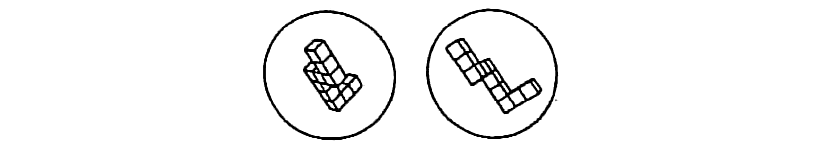


Now, let’s do some sample problems. For each problem, there is a primary object on the far left. You are to determine which two of four objects to the right are the same as the object given on the far left. In each problem, two of the four drawings are the same object as the one on the left. You are to put Xs in the boxes below the correct ones and leave the incorrect ones blank. The first sample problem is done for you.


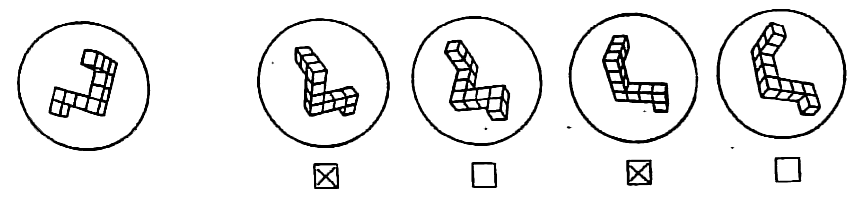


**GO TO THE NEXT PAGE.**

Adapted by S.G. Vandenberg, University of Colorado, July 15, 1971

Revised instructions by H. Crawford, U. of Wyoming, September, 1979

Images digitized and reprinted by Susana Douglas, University of Texas, March, 1996

*This is a public domain document and does not require copyright permission.*

Do the rest of the sample problems yourself. Which two drawings of the four on the right show the same object as the one on the left? There are ALWAYS ONLY two correct answers for each problem. Put an X under the two correct drawings.


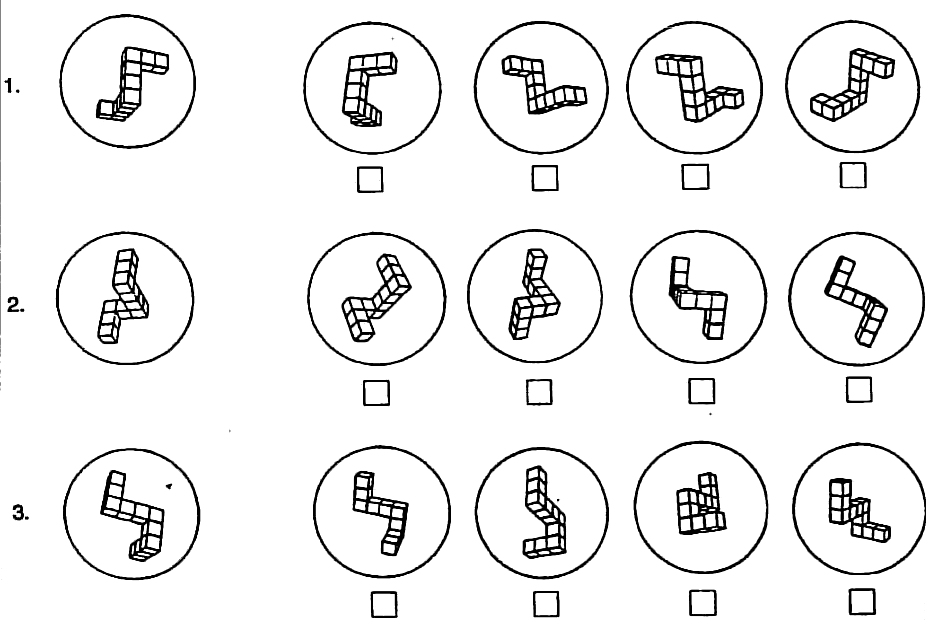


Answers:

1. First and second drawings are correct
2. First and third drawings are correct
3. Second and third drawings are correct

This test has two parts. You will have 5 minutes for each of the two parts. Each part has two pages. When you have finished Part I, STOP. Please do not go on to Part 2 until you are asked to do so. Remember: There are always two and only two correct answers for each item.

Work as quickly as you can without sacrificing accuracy. Your score on this test will reflect both the correct and incorrect responses. Therefore, it will not be to your advantage to guess unless you have some idea which choice is correct.

**DO NOT TURN THIS PAGE UNTIL ASKED TO DO SO.**

**Study ID:** *­­­­­­_________________________*

**PART I**


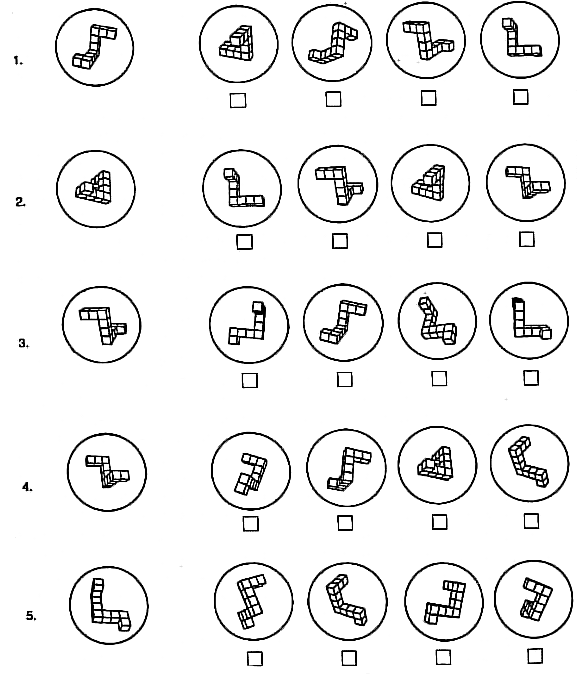


**GO ON TO NEXT PAGE**


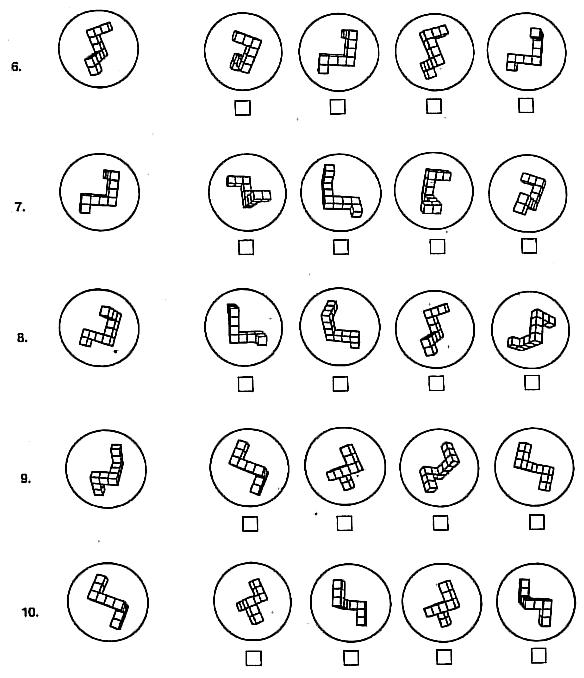


**DO NOT TURN THIS PAGE UNTIL ASKED TO DO SO. STOP**

**Study ID:** *­­­­­­_________________________*

**PART II**


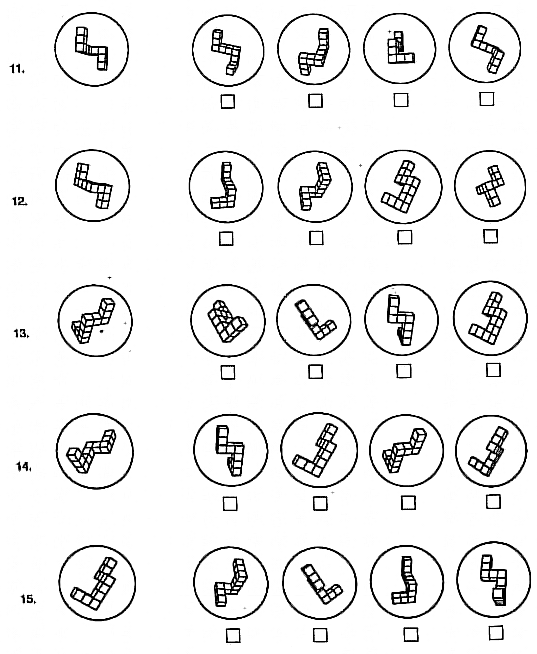


**GO ON TO NEXT PAGE**


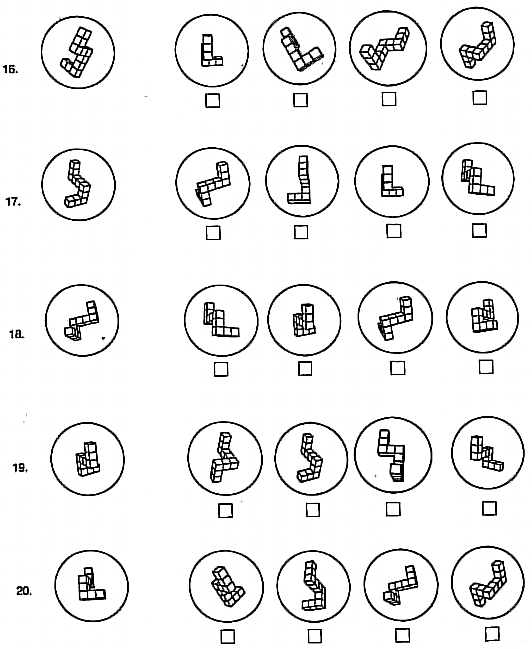


**DO NOT TURN THIS PAGE UNTIL ASKED TO DO SO. STOP**

**Supplemental Figure 4.** Mental Rotations Test (MRT) questionnaire.

**Supplemental Table 1.** Overview of mean ID and pBiS data between modalities. Parameters were calculated to either include all bellringer questions (in.) or exclude (ex.) bellringer questions where pBiS<0.30.

| **Modality** |  | **Mean ID ± SEM** | **Mean pBiS ± SEM** |
| --- | --- | --- | --- |
| **VR** | in. | 0.4611 **±** 0.0485 | 0.5216 **±** 0.0299 |
|  | ex. | 0.4728 **±** 0.0491 | 0.5427 **±** 0.0222 * |
| **Physical** | in. | 0.4767 **±** 0.0471 | 0.5941 **±** 0.0341 |
|  | ex. | 0.4367 **±** 0.0418 | 0.6342 **±** 0.0217 * |
| **2D** | in. | 0.4911 **±** 0.0473 | 0.5263 **±** 0.0398 |
|  | ex. | 0.4836 **±** 0.0445 | 0.5780 **±** 0.0316 |

*Statistically significant differences exist between VR and physical when excluding invalid questions in calculations. ID: item/question difficulty. pBiS, point biserial. Physical: human donor specimen. VR: three-dimensional reconstruction of the physical specimen presented in a virtual reality headset. 2D: high-resolution printed photograph of the physical specimen on paper. SEM: Standard Error of the Mean.
